# Glycoprotein M6B suppresses the maintenance of glioma stem cell stemness and proliferation via the integrin β1/β-catenin pathway

**DOI:** 10.3389/fmolb.2025.1731116

**Published:** 2025-12-10

**Authors:** Yanwen Chen, Yu Fan, Xingda Run, Maotang Liu, Shiwei Jiang, Zengli Miao, Wei Tian

**Affiliations:** 1 Department of Neurosurgery, Jiangnan University Medical Center (Wuxi No. 2 People’s Hospital), Wuxi, Jiangsu, China; 2 Wuxi School of Medicine, Jiangnan University, Wuxi, Jiangsu, China; 3 Wuxi No. 2 People’s Hospital, Affiliated Wuxi Clinical Medical College of Nantong University, Nantong, Jiangsu, China

**Keywords:** glioma stem cells, GPM6B, integrin β1, SOCS3, proliferation

## Abstract

**Background:**

Gliomas are highly aggressive intracranial tumors associated with poor prognosis. Glioma stem cells (GSCs) have been shown to play a pivotal role in tumor progression. Previous studies indicate that GPM6B expression correlates with glioma grade and neuronal differentiation. However, the precise mechanisms of GPM6B in glioma remain unknown. In this study, we aim to elucidate the regulatory role of GPM6B in glioma.

**Methods:**

The relationship between the expression of GPM6B and glioma was analyzed using the CGGA and TCGA databases. Western blot analysis was performed to further validate GPM6B expression in clinical samples. The interaction between GPM6B and the Wnt signaling pathway was explored using co-immunoprecipitation, mass spectrometry, quantitative real-time PCR, and Western blot. These findings were further confirmed in cells transduced with different lentiviral constructs. Tumor sphere formation and extreme limiting dilution assays were conducted to assess the stemness of glioma stem cells. Finally, the role of GPM6B in glioma progression was validated in nude mouse models.

**Results:**

The expression of GPM6B was negatively correlated with glioma grade and prognosis. GPM6B was co-localized with Integrin β1 on the cell membrane. The interaction between GPM6B and Integrin β1 inhibited the expression of β-catenin and its downstream proteins, including p-STAT3, c-Myc, and SOCS3. Moreover, SOCS3 promoted the degradation of GPM6B. Overexpression of GPM6B suppressed tumor sphere formation and reduced the sphere formation efficiency of glioma stem cells. In addition, GPM6B overexpression markedly inhibited the tumorigenic effects of β-catenin and SOCS3. These findings were confirmed *in vivo* experiments.

**Conclusion:**

The expression of GPM6B was negatively correlated with the grade and prognosis of glioma. GPM6B promoted the transformation of glioma stem cells and inhibited growth of glioma by suppressing Integrin β1–mediated regulation of β-catenin, while reducing its own degradation through inhibition of the ubiquitinase SOCS3, thereby stabilizing its function in glioma. Collectively, these results identify GPM6B as a critical regulator of glioma stemness and a potential therapeutic target for glioma, providing new insights into glioma biology and offering a foundation for future translational research.

## 1 Introduction

Gliomas are a type of malignant tumors originating from glial cells of the central nervous system, which are characterized by high invasiveness and aggressiveness, and represent the most common primary intracranial malignant tumors (Weller et al., 2024). With advances in medical technology, the treatment of gliomas has gradually evolved into a comprehensive approach that combines surgery with adjuvant radiotherapy and chemotherapy. Nevertheless, challenges such as poor overall prognosis, high recurrence rates, and resistance to radiotherapy and chemotherapy remain to be resolved. Glioma stem cells (GSCs) are a subpopulation of tumor cells with stem cell–like properties, including self-renewal and multipotent differentiation, and have been shown to contribute to tumor recurrence, neovascularization, and resistance to radiotherapy and chemotherapy (Bao et al., 2006; Agosti et al., 2024; Maleszewska et al., 2024). Elucidating the regulatory mechanisms of GSCs will provide new insights into glioma biology and may lead to the identification of novel therapeutic targets.

Glycoprotein M6B (GPM6B) is a four-transmembrane protein belonging to the proteolipid protein family, and is widely expressed in neurons, oligodendrocytes, and astrocytes (Fjorback et al., 2009; Choi et al., 2013). Previous studies have reported that GPM6B regulates 5-hydroxytryptamine (5-HT) uptake and reduces 5-HT absorption in prostate cancer cells, thereby inhibiting tumor cell proliferation (Fjorback et al., 2009; He et al., 2020). GPM6B also plays an important role in osteoblast differentiation and smooth muscle cell differentiation (Drabek et al., 2011; Zhang et al., 2019). In the nervous system, Bayat et al. (2024) demonstrated that GPM6B promotes the differentiation of NT2 cells into neurons. Aberrant expression of GPM6B may be associated with certain psychiatric disorders. For example, the expression of GPM6B in the hippocampus is decreased in depressed patients who committed suicide (Fuchsova et al., 2015). In addition, genetic variations in GPM6B have been linked to altered phenotypes in certain psychiatric disorders (Sanchez-Roige et al., 2022). As a transmembrane protein, GPM6B participates in multiple physiological and pathological processes, and is particularly relevant to the nervous system and tumor biology. With further investigation, GPM6B may emerge as an important molecular target for neurological disorders and cancer therapy; however, its precise mechanism of glioma remains unclear.

With the increasing focus on glioma stem cells (GSCs), accumulative evidence has demonstrated that the maintenance of GSC stemness is tightly regulated by the activation of multiple signaling pathways, which include the Wnt and Notch pathways. The Wnt pathway is a classical signaling cascade in which integrin β1, upon ligand binding, transduces intracellular signals to activate a series of enzymatic cascades such as the Wnt/β-catenin and PI3K/AKT pathways, thereby regulating cell proliferation and tumor growth (Wang et al., 2019; Liu et al., 2024). Among these, the canonical Wnt/β-catenin pathway has been shown to play a central role in the regulation of GSC stemness. For instance, hypoxic conditions in gliomas induce GLT8D1 expression, which prevents CD133 degradation and promotes Wnt pathway activation, ultimately stabilizing the stem-like properties of GSCs (Liu et al., 2022). In contrast, PER2 overexpression inhibits Wnt/β-catenin pathway activation and consequently attenuates GSC stemness (Ma et al., 2020). In addition, FAM129A has been reported to enhance Notch pathway activation by suppressing NICD1 ubiquitination and degradation, thereby contributing to GSC maintenance (Liu et al., 2023). Our preliminary studies revealed that GPM6B expression is closely associated with the maintenance of GSC stemness (Miao et al., 2022). However, the precise regulatory mechanisms of GPM6B in this process remain largely unknown. Elucidating the mechanism is essential for understanding GSC biology, because it may provide novel therapeutic targets for glioma.

## 2 Materials and methods

### 2.1 Data acquisition and analysis

RNA-seq data and survival information of glioma patients from the TCGA_GBMLGG (n = 669) and CGGA (n = 1,018) databases were downloaded from GlioVis (http://gliovis.bioinfo.cnio.es). The cutoff values used to define high and low GPM6B expression groups were 7.91 for the CGGA dataset and 14.46 for the TCGA dataset. Clinical samples were obtained from Jiangnan University Medical Center (Wuxi No. 2 People’s Hospital). All patients provided informed consent, and the study was approved by the ethics committee (No. 2024Y-107). Gene Ontology (GO) and Genomes (KEGG) pathway analysis were conducted using Sangerbox (http://sangerbox.com/home.html).

### 2.2 Cell acquisition and culture

The acquisition method of GSC cell lines (MES 28, MES 505) was based on previous studies (Miao et al., 2022). GSCS were cultured in DMEM/F12 medium (Gibco, United States) supplemented with B27, basic fibroblast growth factor (bFGF), and epidermal growth factor (EGF, 20 ng/mL). Cells were cultured at 37 °C in a controlled humidified environment with 5% CO_2_.

### 2.3 Quantitative real-time PCR

According to the manufacturer’s instructions, total RNA was extracted from collected MES 28 and MES 505 cells using TRIzol reagent (Thermo Fisher Scientific, United States). The Primer sequences were as follows: GPM6B forward: 5′-AGTACCGTCCTGTGCCAAC-3′ and reverse: 5′-CACAGAACA AGGCCACTCC-3’. Integrin β1 forward: 5′-CCCGAGACCAACCGAGAA-3′ and reverse: 5′-TCCAGGAAACCAGTTGCAAAT-3. SOCS3 forward: 5′- AAGGCCGGAGATTTCGCTT-3′ and reverse: 5′-CGGGAAACTTGCTGTGGG-3’. β-actin forward: 5′- TCTTTGCAGCTCCTTCGT TG-3′ and reverse: 5′-ACGATGGAGGGGAATACAGC-3′.

### 2.4 Western blot

Protein extraction and analysis were performed as previously reported (Zhou et al., 2021). The primary antibodies from Proteintech Group included β-catenin (51067-2-AP, 1:10000), STAT3 (60199-1-Ig, 1:500), p-STAT3 (60479-1-Ig, 1:10000), c-Myc (67447-1-Ig, 1:10000), SOCS3 (14025-1-AP, 1:2000), β-actin (66009-1-Ig, 1:5000). GPM6B was from Antibodies (ABIN1387298, 1:2000). Integrin β1 was from Thermo Fisher Scientific (14-0299-37, 1:1000).

### 2.5 Tumor sphere formation assay

Cells 1 × 10^3^/well were seeded in ultralow-attachment 12-well and were allowed to form spheres over 1 weeks. We acquired images of the spheres using a microscope.

### 2.6 Extreme limiting dilution assay

Transfected GSCs were plated in 96-well plates at densities of 5, 10, 15, 20, 25, 30, 35, 40, 45, 50, 55 and 60 cells per well. Each well was examined for tumor sphere formation after 7 days. Extreme Limiting Dilution Analysis (ELDA) software was used to analyze these data.

### 2.7 Co-immunoprecipitation (Co-IP)

Cells were lysed using RIPA lysis buffer containing protease inhibitor PMSF. The supernatant of the lysate was incubated with the corresponding primary antibody at 4 °C overnight for immunoprecipitation. After washing with 1× loading buffer to eliminate unbound proteins, the precipitated proteins were subjected to immunoblot analysis. Mouse IgG was used as a negative control.

### 2.8 Vectors and lentiviral transfection

The lentiviral vectors designed to knock down Integrin β1 and β-catenin in GSCs was obtained from Genepharma company. The overexpression lentiviral vector for GSCs targeting GPM6B, β-catenin, and SOCS3 were also obtained from Genepharma and verified by DNA sequencing.

### 2.9 Immunofluorescence staining

The tissues were incubated with 3% hydrogen peroxide (H_2_O_2_) for 20 min and blocked with 5% bovine serum albumin (BSA) for 30 min. The samples were subsequently incubated overnight at 4 °C with anti-GPM6B (ABIN1387298, 1:400, Antibodies) and anti-Integrin β1 (14-0299-37, 1:100, Thermo Fisher Scientific) primary antibodies. After washing, they were stained with the secondary antibodies iF555-Tyramide (Cat. No. G1233; Servicebio) and iF647-Tyramide (Cat. No. G1232; Servicebio) at room temperature for 1 h. Nuclei were stained with DAPI (Cat. No. G1012, Servicebio). The microscope was used to acquire images.

### 2.10 Mass spectrometry

Liquid chromatography (LC) coupled with tandem mass spectrometry (MS) was performed by BGI Tech Solutions Co., Ltd. (BGI Shenzhen, Guangdong, China).

### 2.11 Establishment of intracranial nude mouse model

For the intracranial tumor formation assays, GSCs of luciferase-expressing were transplanted into the frontal subdural region of nude mice. An IVIS imaging system be applied to monitor every 7 days tumor growth in the brain. All animal experiments were approved by the Ethics Committee of Jiangnan University (JN. No20250615b0240730 [366]).

### 2.12 Statistical analysis

Statistical analyses were showed using the Prism 10.1.2 software (GraphPad Software, United States). Differences between groups were compared using a Student’s t-test or one-way analysis of variance (ANOVA). Results are shown as mean ± SD and represent results from at least three independent experiments. Statistical significance was defined as P < 0.05.

## 3 Results

### 3.1 The expression of GPM6B is associated with glioma grade and prognosis

**FIGURE 1:**
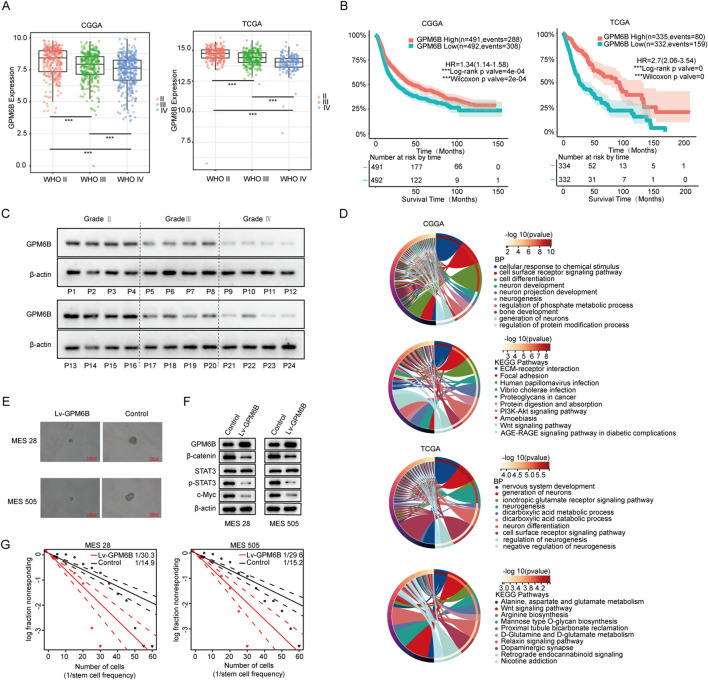
GPM6B expression and function in glioma. **(A)** The expression differences of GPM6B in different grades of glioma. ***P < 0.001. **(B)** The effect of GPM6B expression on patient survival. **(C)** GPM6B expression in different grades of glioma was evaluated by Western blot. **(D)** GO analysis and KEGG pathways analysis of GPM6B. **(E)** Representative tumor sphere images of MES 28 and MES 505 when transfected with Lv-GPM6B, Scale Bar: 100 uM. **(F)** Western blot for GPM6B, β-catenin, STAT3, p-STAT3, and c-Myc levels in MES 28 and MES 505 when treated with Lv-GPM6B. **(G)** *In vitro* limiting dilution assay of MES 28 and MES 505 transduced with vector or Lv-GPM6B.

Analysis of the Chinese Glioma Genome Atlas (CGGA) data revealed that the expression level of GPM6B in gliomas decreased with the increase in grade of glioma (Figure 1A). We also found a significant correlation between GPM6B expression and the prognosis of glioma patients, because patients who exhibit high GPM6B expression showing markedly longer overall survival than those with low expression (Figure 1B). To avoid data bias, we further analyzed glioma sample data from The Cancer Genome Atlas (TCGA) in the United States and obtained similar results (Figures 1A,B). This is consistent with previous studies, showing that GPM6B expression decreases with the increase in grade (Miao et al., 2022). Further detection in clinical glioma samples confirmed that GPM6B expression is lower in high-grade glioma (Figure 1C). GO analysis results indicated that the genes related to GPM6B are mainly involved in neuron development and neuron differentiation in both datasets. KEGG analysis of related genes revealed that GPM6B is associated with the Wnt pathway (Figure 1D). Therefore, we further focus on the role of GPM6B in glioma stem cells. In tumor sphere formation assay, the diameter of spheres in the GPM6B overexpression group was smaller than that in the control group, and the expression of β-catenin, p-STAT3, and c-Myc also reduced (Figures 1E,F). Extreme limiting dilution assay results indicated that higher GPM6B levels would lead to lower rates of stem-cell sphere formation (Figure 1G).

### 3.2 GPM6B can interact with integrin β1 in Wnt/β-catenin pathway

**FIGURE 2:**
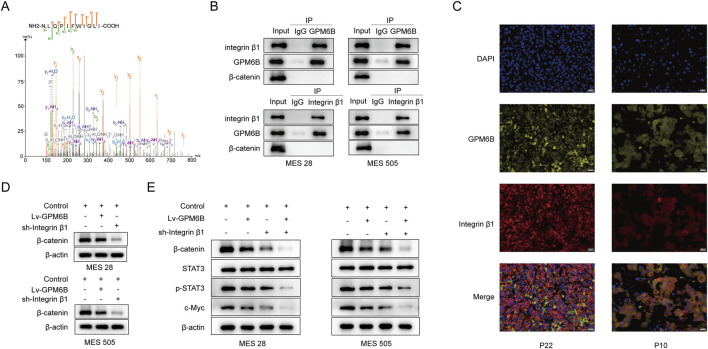
GPM6B can interact with Integrin β1 and inhibits the expression of β-catenin. **(A)** Mass spectrometric sequencing of Integrin β1. **(B)** The interaction between GPM6B and Integrin β1 was confirmed by co-immunoprecipitation in MES 28 and MES 505. **(C)** Images showed that colocalization of GPM6B (yellow) and Integrin β1 (red). Nuclei were counterstained with DAPI (blue). Scale bars: 20 um. **(D)** Western blot for β-catenin levels in MES 28 and MES 505 treated with Lv-GPM6B or sh-Integrin β1. **(E)** Western blot for β-catenin, STAT3, p-STAT3, and c-Myc levels in MES 28 and MES 505 transfected with Lv-GPM6B or sh-Integrin β1.

KEGG analysis of related genes revealed that GPM6B is associated with the Wnt pathway, therefore we would study the relationship between GPM6B and Wnt/β-catenin signaling pathway. We performed immunoprecipitation of proteins from glioma stem cells which are followed by mass spectrometry analysis to identify proteins interacting with GPM6B (Figures 2A,B). The results showed that GPM6B can interact with Integrin β1. Using immunofluorescence staining, we observed co-localization of GPM6B and Integrin β1 on the cell membrane in our clinical samples (Figure 2C).

### 3.3 The GPM6B inhibits the expression of β-catenin

To further investigate the molecular regulatory mechanism of GPM6B in Wnt/β-catenin pathway. We found that GPM6B overexpression or knockdown of the Integrin β1 can inhibit the expression of β-catenin (Figure 2D). We examined the expression levels of wnt-related proteins by Western blot analysis. The results showed that overexpression of GPM6B or knockdown of Integrin β1 led to decreased levels of β-catenin, p-STAT3, and c-Myc, with Integrin β1 knockdown causing a more pronounced reduction compared with GPM6B overexpression (Figure 2E).

### 3.4 β-catenin can regulate the expression of SOCS3

**FIGURE 3:**
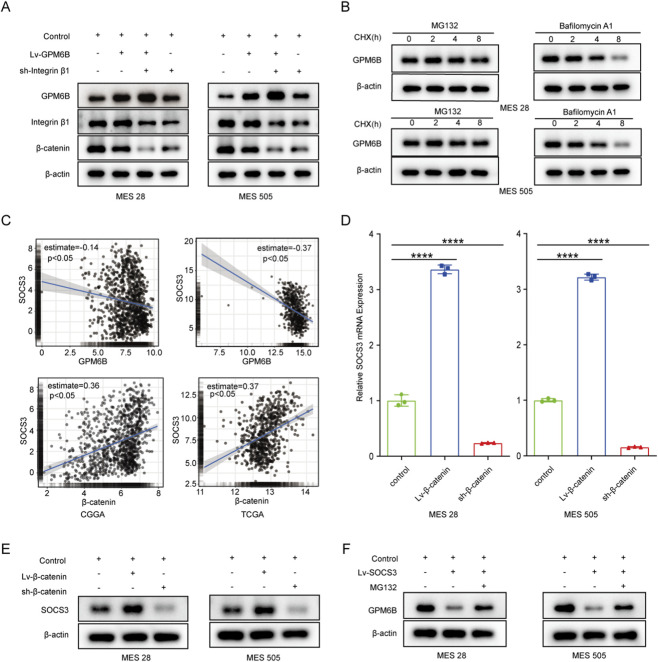
β-catenin can regulate the protein level of GPM6B by modulating the expression of SOCS3. **(A)** Western blot for GPM6B, Integrin β1 and β-catenin levels in MES 28 and MES 505 treated with Lv-GPM6B or sh-Integrin β1. **(B)** The expression of GPM6B in MES 28 or MES 505 when pretreated with MG132 (20 uM) or Bafilomycin A1 (20 uM) and then cells were treated with cycloheximide (100 ug/mL) for an additional 2, 4, and 8 h. **(C)** The correlation between SOCS3 and GPM6B or β-catenin in the CGGA and TCGA datasets. **(D)** qRT-PCR for SOCS3 levels in MES 28 and MES 505 transfected with Lv-β-catenin or sh-β-catenin. Data are indicated as the mean ± SD. ****P < 0.0001. **(E)** Western blot for SOCS3 levels in MES 28 and MES 505 transfected with Lv-β-catenin or sh-β-catenin. **(F)** Western blot for GPM6B levels in MES 28 and MES 505 transfected with Lv-SOCS3 or treated with MG132.

Interestingly, we observed that knockdown of Integrin β1 expression did not alter the mRNA level of GPM6B but increased its protein level, whereas overexpression of GPM6B did not affect either the mRNA or protein levels of Integrin β1. In addition, the expression of β-catenin was regulated by Integrin β1 and GPM6B (Figure 3A; Supplementary Figure S1). In cells, protein degradation primarily occurs through either the lysosomal pathway or the ubiquitin–proteasome pathway. We used cycloheximide (CHX, 100 ug/mL) to inhibit protein synthesis and measured GPM6B protein levels at 0, 2, 4, and 8 h in the present of carbobenzoxy-L-leucyl-L-leucyl-L-leucinal (MG132, 20 uM) or Bafilomycin A1 (20 uM). The results revealed that treatment with the proteasome inhibitor MG132 markedly attenuated the degradation of GPM6B, but treatment with the Bafilomycin A1 had no significant effect on the degradation of GPM6B (Figure 3B). The finding indicates that GPM6B is primarily subjected to proteasomal degradation in glioma stem cells. To identify potential ubiquitinases of regulating GPM6B, we screened the CGGA and TCGA databases. Then, we found that SOCS3 expression was markedly positively connected with the expression of β-catenin and was negatively correlated with the expression of GPM6B (Figure 3C). To further validate this result, we assessed SOCS3 mRNA and protein levels by following β-catenin overexpression or knockdown by using qRT-PCR and Western blot. The results showed that overexpression of β-catenin markedly increased SOCS3 mRNA and protein levels, whereas knockdown of β-catenin significantly decreased SOCS3 mRNA and protein levels (Figures 3D,E). Collectively, these results suggested that β-catenin modulates SOCS3 expression.

### 3.5 SOCS3 promotes the ubiquitin-mediated degradation of GPM6B protein

**FIGURE 4:**
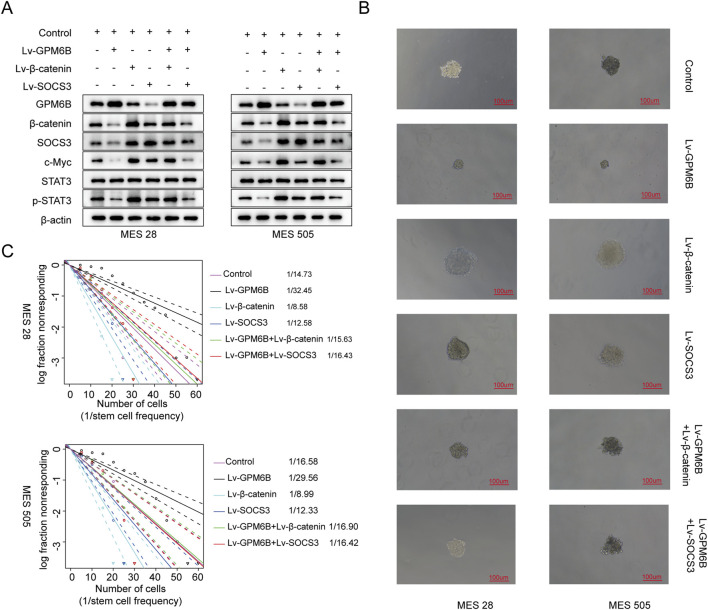
The expression of related proteins in glioma and the influence on cell function under different treatment conditions. **(A)** Western blot for GPM6B, β-catenin, SOCS3, c-Myc, STAT3 and p-STAT3 levels in MES 28 and MES 505 treated with Lv-GPM6B, Lv-β-catenin or Lv-SOCS3. **(B)** Representative tumor sphere images of MES 28 and MES 505 transfected with Lv-GPM6B, Lv-β-catenin or Lv-SOCS3. Scale Bar: 100 uM. **(C)** *In vitro* limiting dilution assay of MES 28 and MES 505 transduced with Lv-GPM6B, Lv-β-catenin or Lv-SOCS3.

We further investigated whether GPM6B is regulated by SOCS3. *In vitro*, we found that the protein level of GPM6B was lower in the SOCS3-overexpressing group than in the controlling group, whereas treatment with the proteasome inhibitor MG132 led to an increase in GPM6B expression (Figure 3F). Moreover, GPM6B overexpression suppressed the expression of β-catenin, SOCS3, c-Myc, and p-STAT3; overexpression of β-catenin or SOCS3 promoted the upregulation of these proteins; whereas GPM6B overexpression in the context of upregulation of β-catenin or SOCS3 markedly attenuated the promoting effect of β-catenin or SOCS3 (Figure 4A). Sphere formation assay and limiting dilution assay of glioma stem cells further validated the above results (Figures 4B,C).

### 3.6 The role of the GPM6B-Integrin β1-β-catenin-SOCS3 signaling axis *in vivo*

**FIGURE 5:**
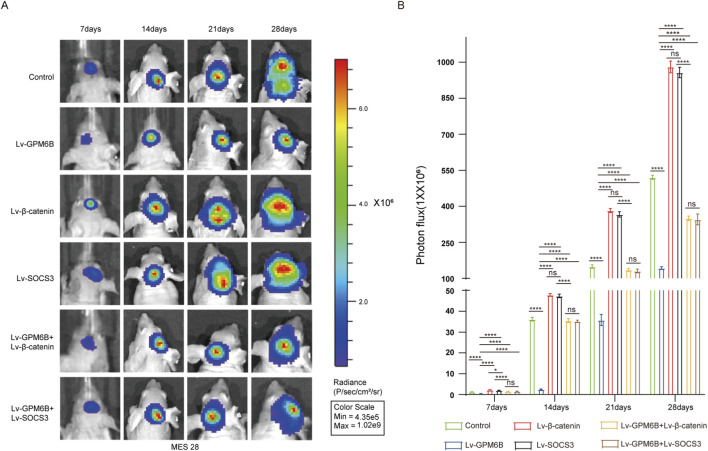
GPM6B inhibits tumor growth *in vivo*. **(A,B)** Bioluminescence imaging was conducted at 7-day intervals after implantation (days 7, 14, 21, and 28). Data expressed as mean ± SD, n = 5 per group. *P < 0.05, ****P < 0.0001.

To verify the role of the GPM6B *in vivo*, we employed an intracranial glioma nude mouse model to investigate its effects on glioma development. Using Bioluminescence imaging, we established different experimental groups and dynamically monitored and recorded the intracranial growth of glioma stem cells. Results showed that GPM6B overexpression suppressed the intracranial growth of glioma stem cells in mice. Overexpression of β-catenin or SOCS3 promoted glioma stem cell growth, whereas GPM6B overexpression in the context of upregulation of β-catenin or SOCS3 markedly attenuated the promoting effect of β-catenin or SOCS3 (Figures 5A,B).

## 4 Discussion

Glioma is one of the most aggressive brain tumors, with limited efficacy of conventional therapies and poor long-term prognosis (Mao et al., 2024). Previous studies have demonstrated that the presence of glioma stem cells is closely associated with the malignant phenotype of gliomas and plays a critical role in tumor recurrence and radioresistance (Biserova et al., 2021; Zhu et al., 2023). Therefore, elucidating the regulatory mechanisms of glioma stem cells is of urgent importance.

GPM6B, a four-transmembrane protein of the proteolipid protein family, is located on Xp22.2 (Feng et al., 2024). It functions through interactions with various proteins; for instance, GPM6B associates with TβRI to promote smooth muscle differentiation (Zhang et al., 2019; Zhu et al., 2020). In recent years, the antitumor effect of GPM6B has been extensively studied. In breast cancer and bladder cancer, patients with high GPM6B expression usually have a better prognosis (Zhu et al., 2020; Mo et al., 2022). In addition, a recent study revealed that GPM6B can inhibit the progression of lung adenocarcinoma (Li et al., 2025). Therefore, it is necessary to investigate the role of GPM6B in glioma. Our previous research has shown that the expression of GPM6B is closely related to the maintenance of GSC stemness (Miao et al., 2022). However, its specific mechanism remains unclear. Therefore, in this study, we further investigated its function and preliminarily revealed its potential mechanism of action.

In this study, we found that the expression level of GPM6B decreased significantly with the increase in grade of glioma in both the CGGA and TCGA databases. Moreover, comparing with those in low expression, patients with high GPM6B expression exhibited markedly longer overall survival. This indicates that GPM6B expression is associated with the malignant phenotype and prognosis of glioma. In clinical specimens, we further observed that GPM6B expression correlated with glioma grade. To investigate the function of GPM6B, we performed GO analysis to GPM6B related genes in CGGA and TCGA datasets, we found they are involved in neuron development and neuron differentiation. Followed by KEGG analysis, the result revealed that they were both in associated with the Wnt pathway. Numerous studies have demonstrated that the WNT signaling pathway plays an important role in tumor stem cell transformation and tumor morphologic transition (Tang et al., 2020; Xue et al., 2024; Yu et al., 2024). Thus, our future studies will focus on determining whether GPM6B mediates glioma stem cell transformation via the WNT signaling pathway. We examined the role of GPM6B in glioma stem cells and found that GPM6B suppressed the sphere-forming capacity of GSCs, accompanied by reduced expression of Wnt pathway–related proteins, including β-catenin, p-STAT3, and c-Myc. These findings suggested that GPM6B may participate in glioma stem cell transformation by suppressing the Wnt/β-catenin signaling pathway. On this basis, we further investigated the relationship between GPM6B and the Wnt signaling pathway. GPM6B and Integrin β1 exhibit colocalization on the cell membrane. We found that GPM6B could interact with Integrin β1, these result also be observed in our clinical samples by immunofluorescence staining. GPM6B overexpression inhibited the expression of the key downstream Wnt signaling protein β-catenin. Interestingly, knockdown of Integrin β1 did not alter the mRNA level of GPM6B but led to an increase in its protein level, which suggested that Integrin β1 regulates the stability of GPM6B protein at the post-transcriptional level in glioma stem cells. We further explored the underlying modification mechanism and eventually found that the expression of β-catenin is positively correlated with the expression of the ubiquitin ligase SOCS3. Moreover, SOCS3 overexpression promotes the ubiquitination and degradation of GPM6B. Furthermore, GPM6B can suppress the expression of β-catenin and its downstream proteins (SOCS3, c-Myc, p-STAT3). However, overexpression of either β-catenin or SOCS3 can attenuate the inhibitory effect of GPM6B on β-catenin and its related proteins. These findings were further validated in tumor sphere formation assays and limiting dilution assays. Finally, in an intracranial glioma model in nude mice, we observed that GPM6B overexpression suppressed intracranial glioma growth, and GPM6B significantly inhibit the functions of β-catenin and SOCS3 in promoting glioma development *in vivo*.

Our study indicates that GPM6B suppresses the proliferation and stemness maintenance of glioma through the Integrin β1–β-catenin signaling pathway, although the detailed mechanism of GPM6B interaction with Integrin β1 requires further investigation. Previous studies have shown that lipid rafts are microdomains in the plasma membrane and are regarded as dynamic platforms for signal transduction, promoting receptor aggregation and thereby activating downstream signal cascade reactions (Capozzi et al., 2023; Warda et al., 2025). The Wnt signaling pathway dependent on lrp6 is a typical example (Riitano et al., 2020). Previous study has shown that lipid rafts can act as a transduction platform to mediate the interaction between CD147 (a transmembrane glycoprotein) and Integrin β1, activating the FAK/cortactin pathway (Li et al., 2018). Therefore, as a transmembrane protein, GPM6B may affect the activation state of integrin β1 through lipid rafts, thereby regulating the transduction of β-catenin signals. Furthermore, we found that GPM6B can inhibit tumor proliferation through the Wnt/β-catenin signaling pathway. Interestingly, previous study has found that inhibiting the Wnt/β-catenin signaling pathway can increase autophagic flux in cells and promote apoptosis in glioblastoma (Nàger et al., 2018). The latest research has further confirmed that changes in autophagy flux can alter the balance between survival and apoptosis in glioblastoma cells (Manganelli et al., 2023). Therefore, it is plausible that GPM6B-mediated inhibition of β-catenin signaling may converge with autophagy/apoptosis regulatory networks, further affecting glioma stemness and tumor progression. Future studies investigating whether GPM6B interacts with Integrin β1 through lipid rafts, and whether the Integrin β1–β-catenin signaling pathway functionally intersects with regulatory networks involved in autophagy and apoptosis, will deepen researchers’ understanding of its role in glioma pathogenesis and provide novel theoretical foundations for potential therapeutic strategies.

In a summary, our findings first demonstrated that GPM6B not only suppresses glioma stem cell capacity and glioma growth by inhibiting Integrin β1–mediated regulation of β-catenin, but also reduces its own degradation by repressing SOCS3-mediated ubiquitination, thereby stabilizing itself in glioma. This finding underscores the significant research value of GPM6B in a clinical context and warrants further investigation.

## 5 Conclusion

GPM6B may promote the transformation of glioma stem cells and inhibit growth of glioma by suppressing Integrin β1–mediated regulation of β-catenin, while simultaneously reducing its own degradation through inhibition of the ubiquitinase SOCS3, thereby stabilizing its function in glioma. These findings showed that GPM6B is a potential novel target for glioma therapy, and further investigations will facilitate the translation of this discovery into clinical applications.

## Data Availability

The datasets presented in this study can be found in online repositories. The names of the repository/repositories and accession number(s) can be found in the article/Supplementary Material.
