# Supplementary material for: Glycoprotein M6B suppresses the maintenance of glioma stem cell stemness and proliferation via the integrin β1/β-catenin pathway
Source: Front Mol Biosci. 2025 Dec 10;12:1731116. doi: 10.3389/fmolb.2025.1731116 (PMC12727601; doi:10.3389/fmolb.2025.1731116)
Supplement: Supplementary file 5 [file Image1.pdf]

frontiers

Supplementary Material

1.1 Supplementary Figures

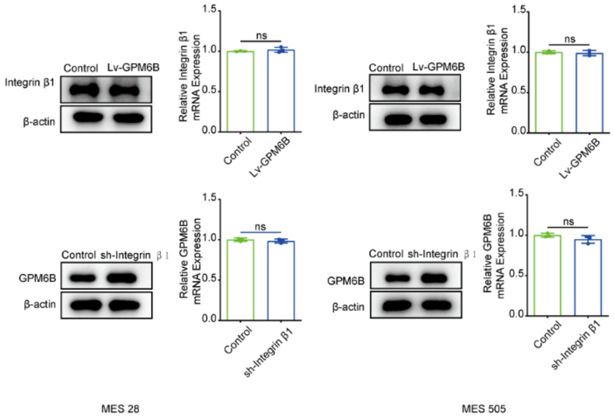

Supplementary Figure S1. Integrin β1 affects the protein level of GPM6B at the post-transcriptional level.

qRT-PCR and Western blot analyses of GPM6B and Integrin β1 expression in MES 28 and MES 505 treated with Lv-GPM6B or sh-Integrin β1. Data are indicated as the mean±SD. *$P < 0.05$。
